# Absolute treatment effects for the primary outcome and all-cause mortality in the cardiovascular outcome trials of new antidiabetic drugs: a meta-analysis of digitalized individual patient data

**DOI:** 10.1007/s00592-022-01917-9

**Published:** 2022-07-25

**Authors:** Oliver Kuss, Cihan Akbulut, Sabrina Schlesinger, Asen Georgiev, Malte Kelm, Michael Roden, Georg Wolff

**Affiliations:** 1 Institute for Biometrics and Epidemiology, German Diabetes Center, Leibniz Center for Diabetes Research at Heinrich-Heine-University Düsseldorf, Düsseldorf, Germany; 2 Centre for Health and Society, Medical Faculty and University Hospital Düsseldorf, Heinrich-Heine-University Düsseldorf, Düsseldorf, Germany; 3 German Center for Diabetes Research, Partner Düsseldorf, München-Neuherberg, Germany; 4 Institute for Clinical Diabetology, German Diabetes Center, Leibniz Center for Diabetes Research at Heinrich-Heine-University Düsseldorf, Düsseldorf, Germany; 5 Division of Cardiology, Pulmonology and Vascular Medicine, Department of Internal Medicine, Medical Faculty and University Hospital Düsseldorf, Heinrich-Heine-University Düsseldorf, Düsseldorf, Germany; 6 Department of Endocrinology and Diabetology, Medical Faculty and University Hospital Düsseldorf, Heinrich-Heine-University Düsseldorf, Düsseldorf, Germany; 7 Deutsches Diabetes-Zentrum, Institut für Biometrie und Epidemiologie, Auf’m Hennekamp 65, 40225 Düsseldorf, Germany

**Keywords:** Number needed to treat, Hazard ratio, Cardiovascular outcome trial, Meta-analysis, Digitalized individual patient data, DPP-4 inhibitors, GLP-1 receptor agonists, SGLT2 inhibitors

## Abstract

**Aims:**

Treatment effects from the large cardiovascular outcome trials (CVOTs) of new antidiabetic drugs are almost exclusively communicated as hazard ratios, although reporting guidelines recommend to report treatment effects also on an absolute scale, e.g. as numbers needed to treat (NNT). We aimed to analyse NNTs in CVOTs comparing dipeptidyl peptidase-4 (DPP-4) inhibitors, glucagon-like peptide-1 (GLP-1) receptor agonists, or sodium–glucose cotransporter-2 (SGLT2) inhibitors to placebo.

**Methods:**

We digitalized individual time-to-event information for the primary outcome and all-cause mortality from 19 CVOTs that compared DPP-4 inhibitors, GLP-1 receptor agonists, or SGLT2 inhibitors to placebo. We estimated Weibull models for each trial and outcome and derived monthly NNTs. NNTs were summarized across all trials and within drug classes by random effects meta-analysis methods.

**Results:**

Treatment effects in the CVOTs appear smaller if they are reported as NNTs: Overall, 100 (95%-CI: 60, 303) patients have to be treated for 29 months (the median follow-up time across all trials) to avoid a single event of the primary outcome, and 128 (95%-CI: 85, 265) patients have to be treated for 39 months to avoid a single death. NNT time courses are very similar for GLP-1 receptor agonists and SGLT2 inhibitors, whereas treatment effects with DPP-4 inhibitors are smaller.

**Conclusions:**

We found that the respective treatment effects look less impressive when communicated on an absolute scale, as numbers needed to treat. For a valid overall picture of the benefit of new antidiabetic drugs, trial authors should also report treatment effects on an absolute scale.

**Supplementary Information:**

The online version contains supplementary material available at 10.1007/s00592-022-01917-9.

## Introduction

In reaction to the “rosiglitazone case” [1], and as requested by the US Food and Drug Administration (FDA) [2], pharmaceutical industry performed a large number of the so called “Cardiovascular Outcome Trials” (CVOTs) in the last decade [3]. Although these trials were primarily focused on showing non-inferiority against placebo, many of the investigated drugs also showed superiority and therefore informed and changed current guidelines for diabetes treatment [4, 5]. As non-inferiority has to be shown on the hazard ratio (HR) scale (with the upper limit of the two-sided 95% confidence interval not exceeding 1.3), CVOTs almost exclusively report these relative effect estimates to describe treatment differences. However, it has long been recognized and recommended in guidelines [6–8] to report treatment effects also on an absolute scale, e.g. as numbers needed to treat (NNT), only recently in the context of CVOTs as well [9]. An NNT gives the number of patients that have to be treated to prevent one additional single event of the outcome in the treatment group. Positive values of the NNT can thus be interpreted in the CVOTs as the treatment being beneficial compared to placebo. A null effect of the treatment, corresponding to a HR of 1, is given by the value of infinity for the NNT. In the CVOTs with their outcomes being time to an event, NNTs are necessarily time dependent, that is, change their values with duration of treatment [10].

Absolute effects should be reported next to relative ones, because treatment effects on relative scales appear more impressive to patients, physicians, and policy makers [11]. In addition and from a more general, philosophical viewpoint, Sprenger/Stegenga [12] point to further deficiencies of relative effect measures. With respect to decision theory, absolute effect measures are more helpful for assessing and maximizing utility of treatments. Concerning causal inference, absolute effect measures naturally combine assessments of causal strength, e.g. when mediators or combined outcomes are involved.

Nevertheless and largely ignoring guidelines, absolute effects are still underreported in the medical literature [13–17] and this is also true for the CVOTs which almost exclusively report hazard ratios in the original publications.

There have been previous analyses of NNTs in the large CVOTs in type 2 diabetes [10, 18] with respect to the trials’ primary outcomes. We extend these analyses in the following by meta-analysing and comparing NNTs for the three different drug classes of DPP-4 inhibitors, GLP-1 receptor agonists, and SGLT2 inhibitors. In addition, we consider the outcome of all-cause mortality and offer NNTs for the trials’ observation times as well as an extrapolation of NNTs to 30 years of treatment.

## Materials and methods

We performed what we propose to call a meta-analysis of “digitalized individual patient data”. We downloaded full texts and online supplements of the CVOTs that (1) were given as completed or ongoing in Fig. 1 of Cefalu et al. [3], (2) had been finished and published until September 2020, and (3) compared a DPP-4 inhibitor, a GLP-1 receptor agonist, or an SGLT2 inhibitor to placebo. We used WebPlotDigitizer, version 4.2, [19] and the R code of Guyot et al. [20] to extract individual patients’ time-to-event information from the Kaplan–Meier plots in the original trial populations. Both methods have been shown to be reliable and valid [21–23].

Data were extracted for the respective trial’s primary outcome and the outcome of all-cause mortality. For calculating absolute treatment effects for time-to-event outcomes in a single trial, it is necessary to estimate the survival functions in both treatment groups. As this is not possible from standard Cox proportional hazard models, we fitted, for each outcome in each trial separately, parametric Weibull regression model for the treatment effect. Weibull models are parametric proportional hazards models [24] and thus yield hazard ratios for the treatment effect which can be compared to the hazard ratios from the original paper. From the respective Weibull model, we estimated monthly probability differences (treatment–control) for being free of the event of interest from month 1 to the respective trial’s maximal observation time. These probability differences were then inverted to arrive at estimates for the monthly number needed to treat. In supplemental Fig. 1, we explain this procedure by showing how to compute the NNT for all-cause mortality after 36 months from the EMPA-REG trial.

In the interest of achieving a lifetime perspective of treatment effects, we additionally projected NNTs until 360 months of treatment. This was done (due to the threat of competing risk by death for all other outcomes) only for the outcome of all-cause mortality and under the assumptions that patients would remain on the same treatment and that the treatment effect remains constant after trial completion.

To assess the validity of the extracted data, we compared hazard ratios from the original papers to those from the Weibull models by calculating intra-class correlation coefficients. In addition, and to assess the fit of the Weibull models graphically, we also plotted Kaplan–Meier estimates from the digitalized data together with predicted survival functions from these models.

To summarize NNTs overall and in the three drug classes, we used random-effects inverse-variance meta-analysis methods. Meta-analyses were calculated separately for each single time point. All computations were performed on the probability difference scale and only for displaying results in figures and graphs transformed to the NNT scale. We used SAS (SAS Institute Inc., Cary, NC, USA), version 9.4, for data management and analysis. The full data set is available in a public repository [25]. As the study does not include personalized data, we did not seek for a vote of an ethics committee. The study was not pre-registered and had no previously published protocol because we were developing the statistical methods in parallel with the analysis.

## Results

Overall we achieved the original time-to-event information from 4 trials on DPP-4 inhibitors (CARMELINA, EXAMINE, SAVOR-TIMI 53, TECOS), 7 trials on GLP-1 receptor agonists (ELIXA, EXSCEL, HARMONY, LEADER, PIONEER, REWIND, SUSTAIN), and 8 trials on SGLT2 inhibitors (CANVAS, CREDENCE, DAPA-CKD, DAPA-HF, DECLARE-TIMI 58, EMPA-REG, EMPEROR-REDUCED, VERTIS-CV). We excluded the CAROLINA trial, because it has no placebo control; the results of two other trials (FREEDOM-CVO, EMPEROR-PRESERVED) in Fig. 1 of Cefalu et al. [3] were not yet available as of September 2020.

Table 1 gives an overview of the included trials with the treatment and drug class under study, a description of the trial populations, and the exact definition of the primary outcome. As reported in Table 2, 19 trials with 17,501 events from 159,265 observations gave information on the primary outcome, and 13 trials with 8,888 events from 112,524 observations on all-cause mortality. Median follow-up times in the trials ranged from 14.1 to 64.9 months for the primary outcome, and from 15.6 to 65.4 months for all-cause mortality. The overall median follow-up time was 28.7 months for the primary outcome and 39.3 months for all-cause mortality. The hazard ratios from the original publications ranged from 0.61 to 1.02 for the primary outcome, and from 0.68 to 1.01 for all-cause mortality, the respective median hazard ratios were 0.87 for both outcomes. The originally reported hazard ratios, together with the computed hazard ratios from the digitalized data (for a Cox and a Weibull model) are also given in Table 2.

**Table 1:** Description of included trials (Abbreviations: SD = standard deviation, BMI = body mass index, CV = cardiovascular, MI = myocardial infarction, ESKD = end-stage kidney disease). When only medians and/or quartiles and/or minima/maxima were reported in the trails, we used the formula of Wan et al. [26] to calculate the respective mean and standard deviations

Trial	Drug	Mean (SD) age (years)	Proportion male (%)	Mean (SD) HbA1c (%)	Mean (SD) BMI (kg/m^2^)	Mean (SD) diabetes duration (years)	Components of primary outcome
*DPP-4 inhibitors*							
CARMELINA	Linagliptin	65.9 (9.1)	62.9	7.9 (1.0)	31.4 (5.3)	14.8 (9.5)	CV death, nonfatal MI, or nonfatal stroke
EXAMINE	Alogliptin	61.0 (–-)	67.8	8.0 (1.1)	33.8 (6.6)	7.7 (8.2)	Death from CV causes, nonfatal MI, or nonfatal stroke
SAVOR-TIMI53	Saxagliptin	65.1 (8.5)	66.9	8.0 (1.4)	31.1 (5.6)	10.7 (8.5)	CV death, MI, or ischemic stroke
TECOS	Sitagliptin	65.5 (8.0)	70.7	7.2 (0.5)	30.2 (5.7)	11.6 (8.1)	CV death, nonfatal MI, nonfatal stroke, or hospitalization for unstable angina
*GLP-1 receptor agonists*							
ELIXA	Lixisenatide	60.3 (9.7)	69.4	7.7 (1.3)	30.2 (5.7)	9.3 (8.3)	CV death, MI, stroke, or hospitalization for unstable angina
EXSCEL	Exenatide	62.0 (8.9)	62.0	8.1 (1.2)	32.0 (5.9)	12.2 (7.8)	Death from CV causes, nonfatal MI, or nonfatal stroke
HARMONY	Albiglutide	64.2 (8.7)	69.4	8.7 (1.5)	32.3 (5.9)	14.2 (8.8)	First occurrence of CV death, MI, or stroke
LEADER	Liraglutide	64.3 (7.2)	64.2	8.7 (1.5)	32.5 (6.3)	12.9 (8.1)	Death from CV causes, nonfatal MI, or nonfatal stroke
PIONEER	Semaglutide	66.0 (7.0)	68.4	8.2 (1.6)	32.3 (6.5)	14.9 (8.5)	Death from CV causes, nonfatal MI, or nonfatal stroke
REWIND	Dulaglutide	66.2 (6.5)	53.7	7.4 (1.1)	32.3 (5.8)	10.6 (7.2)	Nonfatal MI, nonfatal stroke, or death from CV causes
SUSTAIN	Semaglutide	64.6 (7.4)	60.7	8.7 (1.5)	32.8 (6.2)	13.9 (8.1)	CV death, nonfatal MI, or nonfatal stroke
*SGLT2 inhibitors*							
CANVAS	Canagliflozin	63.3 (8.3)	64.2	8.2 (0.9)	31.9 (5.9)	13.6 (7.7)	Sustained and adjudicated doubling in serum creatinine, ESKD, or death from renal causes
CREDENCE	Canagliflozin	63.0 (9.2)	66.0	8.3 (1.3)	31.4 (6.2)	15.7 (8.7)	ESKD, doubling of the serum creatinine level, or death from renal or CV causes
DAPA-CKD	Dapagliflozin	61.9 (12.2)	66.9		29.5 (6.2)		Sustained decline in the estimated GFR of at least 50%, ESKD, or death from renal or CV causes
DAPA-HF	Dapagliflozin	66.3 (10.9)	77.7		28.2 (6.0)		Worsening heart failure or CV death
DECLARE-TIMI 58	Dapagliflozin	63.9 (6.8)	62.6	8.3 (1.2)	32.1 (6.0)	10.9 (7.4)	CV death, MI, or ischemic stroke
EMPA-REG	Empagliflozin	63.1 (8.7)	71.5	8.1 (0.8)	30.6 (5.2)		Death from CV causes, nonfatal MI, or nonfatal stroke
EMPEROR-REDUCED	Empagliflozin	66.8 (11.0)	76.0		27.9 (5.4)		CV death or hospitalization for heart failure
VERTIS-CV	Ertugliflozin	64.4 (8.1)	70.0	8.2 (1.0)	31.9 (5.4)	13.0 (8.3)	Death from CV causes, nonfatal MI, or nonfatal stroke

**Table 2:** Description of included trials, separated by outcomes and drug classes (Abbreviation: HR = hazard ratio, CI = confidence interval)

Trial	Number of events	Number of observations	Event proportion (%)	Median follow-up time (months)	Originally reported HR [95% CI]	HR [95% CI] as computed from the digitalized data	HR [95% CI] as computed from the digitalized data under a Weibull model
*Primary outcome*							
*DPP-4 inhibitors*							
CARMELINA	852	6979	12.2	25.9	1.02 [0.89,1.17]	1.02 [0.90,1.17]	1.02 [0.89,1.16]
EXAMINE	621	5380	11.5	18.4	0.96 [ –,-]	0.96 [0.82,1.12]	0.95 [0.80,1.10]
SAVOR-TIMI53	1217	16,492	7.4	24.8	1.00 [0.89,1.12]	1.00 [0.89,1.11]	1.00 [0.88,1.11]
TECOS	1686	14,671	11.5	34.0	0.98 [0.89,1.08]	0.99 [0.90,1.09]	0.98 [0.88,1.07]
*GLP-1 receptor agonists*							
ELIXA	790	6068	13.0	24.5	1.02 [0.89,1.17]	1.00 [0.87,1.15]	1.01 [0.87,1.15]
EXSCEL	1708	14,752	11.6	35.2	0.91 [0.83,1.00]	0.91 [0.83,1.00]	0.91 [0.83,1.00]
HARMONY	766	9463	8.1	19.0	0.78 [0.68,0.90]	0.79 [0.68,0.91]	0.78 [0.67,0.89]
LEADER	1298	9340	13.9	45.9	0.87 [0.78,0.97]	0.87 [0.78,0.96]	0.87 [0.77,0.96]
PIONEER	135	3183	4.2	15.9	0.79 [0.57,1.11]	0.79 [0.56,1.11]	0.79 [0.52,1.06]
REWIND	1243	9901	12.6	64.8	0.88 [0.79,0.99]	0.88 [0.79,0.99]	0.88 [0.79,0.98]
SUSTAIN	221	3297	6.7	22.1	0.74 [0.58,0.95]	0.73 [0.56,0.95]	0.72 [0.53,0.92]
*SGLT2 inhibitors*							
CANVAS	1036	10,142	10.2	28.9	0.86 [0.75,0.97]	0.86 [0.76,0.97]	0.87 [0.77,0.98]
CREDENCE	583	4401	13.2	30.6	0.70 [0.59,0.82]	0.69 [0.59,0.82]	0.69 [0.58,0.81]
DAPA-CKD	493	4304	11.5	25.5	0.61 [0.51,0.72]	0.60 [0.50,0.72]	0.60 [0.49,0.71]
DAPA-HF	885	4744	18.7	17.5	0.74 [0.65,0.85]	0.75 [0.65,0.85]	0.75 [0.65,0.84]
DECLARE-TIMI58	1447	17,160	8.4	47.3	0.93 [0.84,1.03]	0.94 [0.84,1.04]	0.94 [0.84,1.03]
EMPA-REG	753	7020	10.7	36.2	0.86 [0.74,0.99]	0.85 [0.73,0.98]	0.84 [0.72,0.97]
EMPEROR-REDUCED	808	3730	21.7	14.2	0.75 [0.65,0.86]	0.75 [0.65,0.86]	0.75 [0.65,0.85]
VERTIS-CV	959	8238	11.6	32.8	0.97 [0.85,1.11]	0.95 [0.83,1.09]	0.95 [0.82,1.08]
All-cause mortality							
*DPP-4 inhibitors*							
CARMELINA	739	6979	10.6	26.8	0.98 [0.84,1.13]	0.97 [0.84,1.12]	0.97 [0.83,1.11]
EXAMINE	316	5380	5.9	19.5	0.88 [0.71,1.09]	0.89 [0.72,1.11]	0.89 [0.70,1.09]
TECOS	1042	14,671	7.1	36.2	1.01 [0.90,1.14]	1.01 [0.89,1.14]	1.01 [0.88,1.13]
*GLP-1 receptor agonists*							
EXSCEL	1051	14,752	7.1	40.1	0.86 [0.77,0.97]	0.86 [0.77,0.98]	0.86 [0.76,0.97]
LEADER	824	9340	8.8	46.5	0.85 [0.74,0.97]	0.84 [0.74,0.97]	0.85 [0.73,0.96]
REWIND	1109	9901	11.2	65.4	0.90 [0.80,1.01]	0.89 [0.79,1.00]	0.89 [0.78,0.99]
*SGLT2 inhibitors*							
CANVAS	701	10,142	6.9	29.6	0.87 [0.74,1.01]	0.85 [0.73,0.99]	0.86 [0.73,0.99]
CREDENCE	366	4401	8.3	31.5	0.83 [0.68,1.02]	0.82 [0.67,1.01]	0.82 [0.65,0.99]
DAPA-CKD	234	4304	5.4	27.5	0.69 [0.53,0.88]	0.68 [0.52,0.88]	0.68 [0.50,0.85]
DAPA-HF	598	4744	12.6	18.2	0.83 [0.71,0.97]	0.84 [0.71,0.98]	0.84 [0.70,0.97]
DECLARE-TIMI58	954	17,160	5.6	47.3	0.93 [0.82,1.04]	0.95 [0.84,1.08]	0.95 [0.83,1.07]
EMPA-REG	461	7020	6.6	37.7	0.68 [0.57,0.82]	0.67 [0.56,0.81]	0.67 [0.55,0.80]
EMPEROR-REDUCED	493	3730	13.2	15.6	0.92 [0.77,1.10]	0.91 [0.76,1.09]	0.92 [0.76,1.08]

**Fig. 1:**
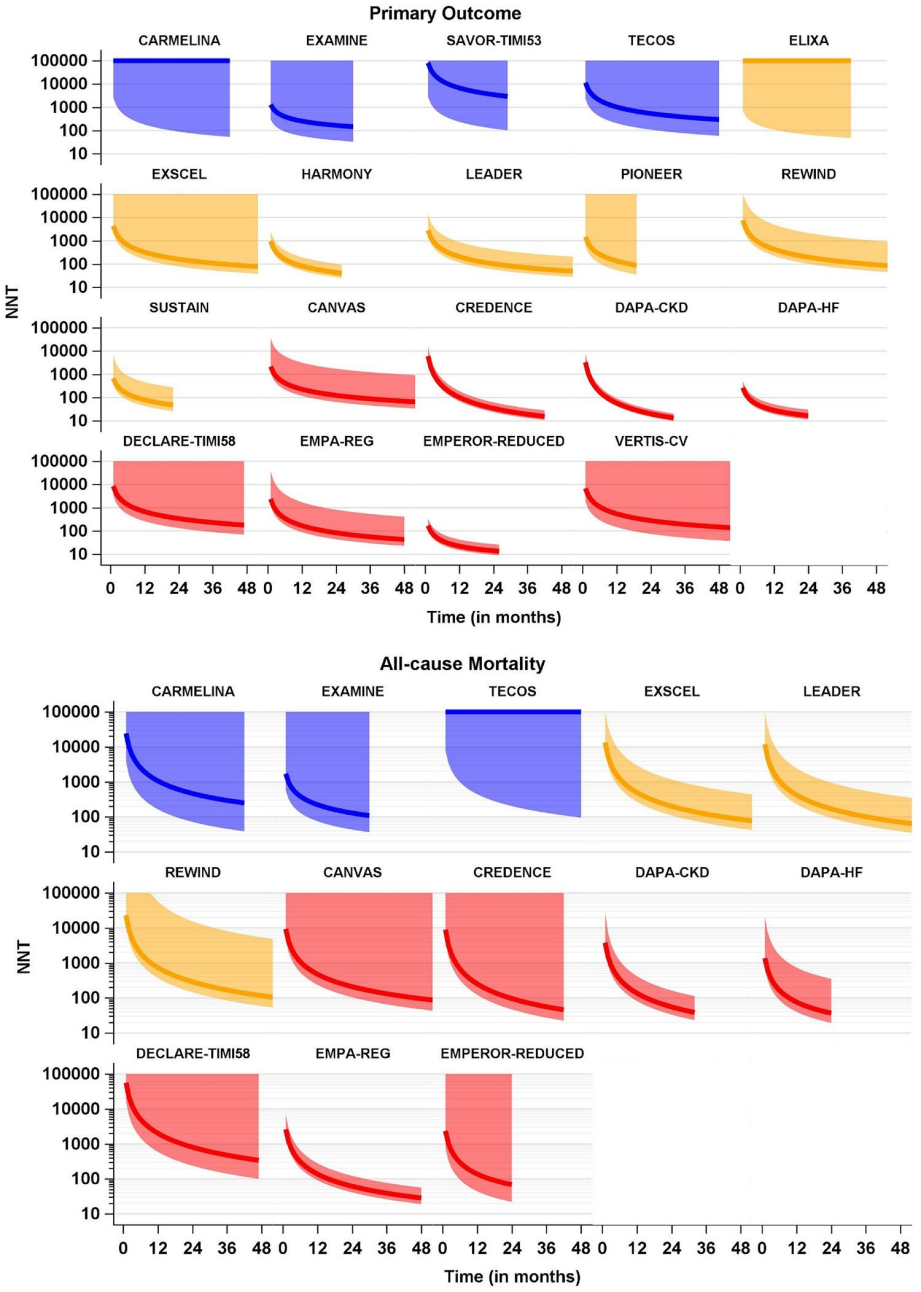
NNTs for the single trials, separated by outcomes and drug classes (blue: DPP-4 inhibitors, yellow: GLP-1 receptor agonists, red: SGLT2 inhibitors) with their pointwise 95% confidence intervals. Estimates and confidence intervals are truncated from above at 100.000. Please note the logarithmic scale on the y-axis (Color figure online)

Figure 1 shows the trials’ NNT time courses for both outcomes, annual NNT values after 12, 24, 36, and 48 months of treatment are also given in Table 3.

**Table 3:** Annual NNTs for years 1, 2, 3, and 4 with 95% confidence intervals, separated by outcomes and drug classes

Trial	NNT [95% CI] after			
	12 months	24 months	36 months	48 months
*Primary outcome*				
*DPP-4 inhibitors*				
CARMELINA	− 898 [181, − 129]	− 446 [90, − 64]	− 304 [62, − 44]	
EXAMINE	255 [58, − 107]	167 [38, − 70]		
SAVOR-TIMI53	6765 [237, − 255]	3487 [122, − 131]		
TECOS	1026 [207, − 347]	540 [109, − 183]	378 [76, − 128]	296 [60, − 100]
*GLP-1 receptor agonists*				
ELIXA	− 1816 [108, − 97]	− 1112 [66, − 59]	− 849 [51, − 45]	
EXSCEL	326 [159, − 7005]	162 [79, − 3577]	110 [54, − 2433]	84 [41, − 1867]
HARMONY	83 [52, 197]	43 [27, 103]		
LEADER	207 [117, 881]	103 [58, 432]	69 [39, 291]	53 [30, 223]
PIONEER	136 [55, − 299]			
REWIND	434 [227, 4938]	198 [104, 2163]	127 [67, 1366]	93 [49, 999]
SUSTAIN	80 [44, 464]			
*SGLT2 inhibitors*				
CANVAS	229 [119, 3170]	125 [65, 1732]	89 [46, 1236]	70 [36, 981]
CREDENCE	103 [70, 191]	35 [24, 63]	19 [13, 35]	
DAPA-CKD	58 [43, 92]	20 [15, 31]		
DAPA-HF	28 [20, 52]	17 [12, 30]		
DECLARE-TIMI58	683 [266, − 1208]	343 [134, − 609]	232 [91, − 412]	
EMPA-REG	170 [89, 1733]	84 [44, 822]	57 [30, 545]	43 [23, 413]
EMPEROR-REDUCED	22 [15, 43]	14 [10, 27]		
VERTIS-CV	549 [147, − 317]	280 [75, − 162]	192 [52, − 111]	149 [40, − 86]
*All-cause mortality*				
*DPP-4 inhibitors*				
CARMELINA	1070 [165, − 239]	467 [72, − 104]	296 [46, − 66]	
EXAMINE	223 [75, − 230]	129 [43, − 133]		
TECOS	− 9238 [445, − 406]	− 4221 [204, − 186]	− 2703 [130, − 119]	− 1988 [96, − 87]
*GLP-1 receptor agonists*				
EXSCEL	497 [271, 2948]	202 [110, 1168]	121 [66, 694]	85 [46, 486]
LEADER	423 [232, 2444]	170 [93, 943]	101 [56, 555]	71 [39, 387]
REWIND	740 [372, 57774]	288 [145, 15415]	168 [85, 8117]	116 [58, 5383]
*SGLT2 inhibitors*				
CANVAS	475 [237,− 96864]	210 [105,− 50993]	131 [66,− 27830]	95 [47,− 17270]
CREDENCE	252 [123, − 5131]	96 [47, − 2356]	56 [27, − 1405]	
DAPA-CKD	137 [82, 419]	55 [33, 163]		
DAPA-HF	76 [40, 735]	37 [19, 353]		
DECLARE-TIMI58	2019 [596, − 1455]	808 [239, − 583]	477 [141, − 344]	
EMPA-REG	137 [91, 280]	62 [41, 122]	39 [26, 77]	28 [19, 56]
EMPEROR-REDUCED	138 [44, -124]	69 [22, − 62]		

**Fig. 2:**
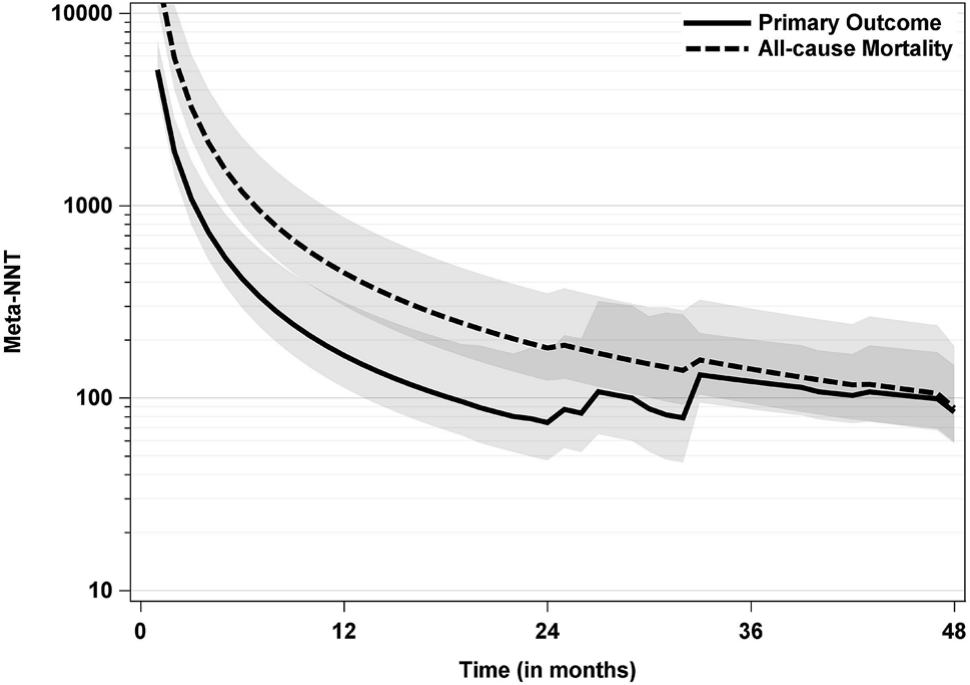
Meta-NNTs for the two outcomes across all trials and drug classes with their pointwise 95% confidence intervals. Meta-NNTs were calculated by standard random-effects inverse-variance meta-analysis methods, separately for each month. All computations were performed on the probability difference scale and only then transformed to the NNT scale. Please note the logarithmic scale on the y-axis. With respect to the primary outcome, Meta-NNTs were computed from the single trials’ primary outcomes which slightly differ in their definition (see Table 1)

The Meta-NNT time course, as summarized across all trials and drug classes, is given in Fig. 2. At the overall median follow-up times of 29 months for the primary outcome and 39 months for all-cause mortality, the estimated Meta-NNTs are 100 (95%-CI: 60, 303) and 128 (95%-CI: 85, 265), respectively.

**Fig. 3:**
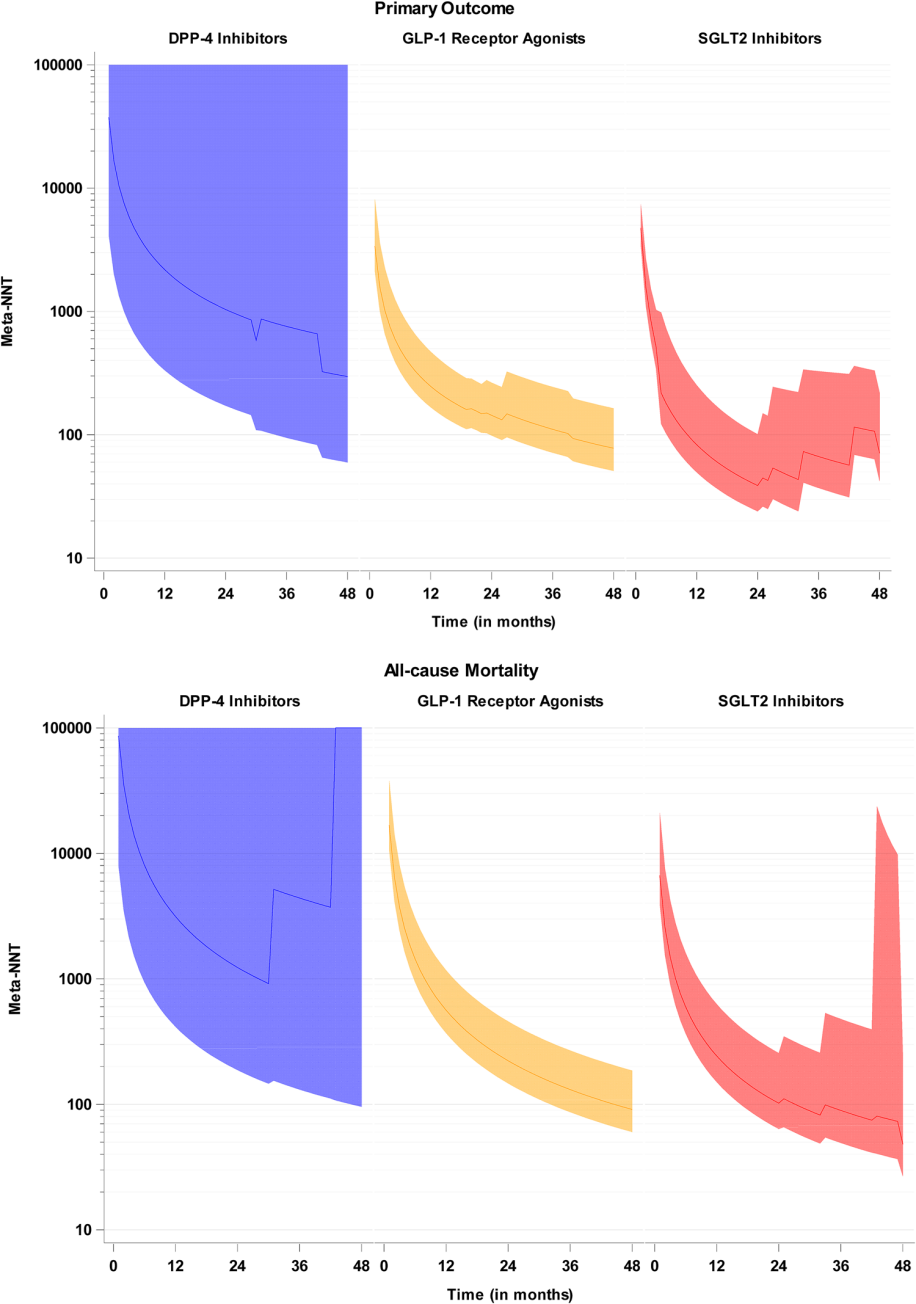
Overall Meta-NNTs for the two outcomes, separated by drug classes (blue: DPP-4 inhibitors, yellow: GLP-1 receptor agonists, red: SGLT2 inhibitors) with their pointwise 95% confidence intervals. Estimates and confidence intervals are truncated from above at 100.000. Please note the logarithmic scale on the y-axis. With respect to the primary outcome, Meta-NNTs were computed from the single trials’ primary outcomes which slightly differ in their definition (see Table 1) (Color figure online)

With respect to Meta-NNTs in the three different drug classes under study (Fig. 3), NNT time courses are very similar with GLP-1 receptor agonists vs. SGLT2 inhibitors, whereas treatment effects with DPP-4 inhibitors are smaller.

**Fig. 4:**
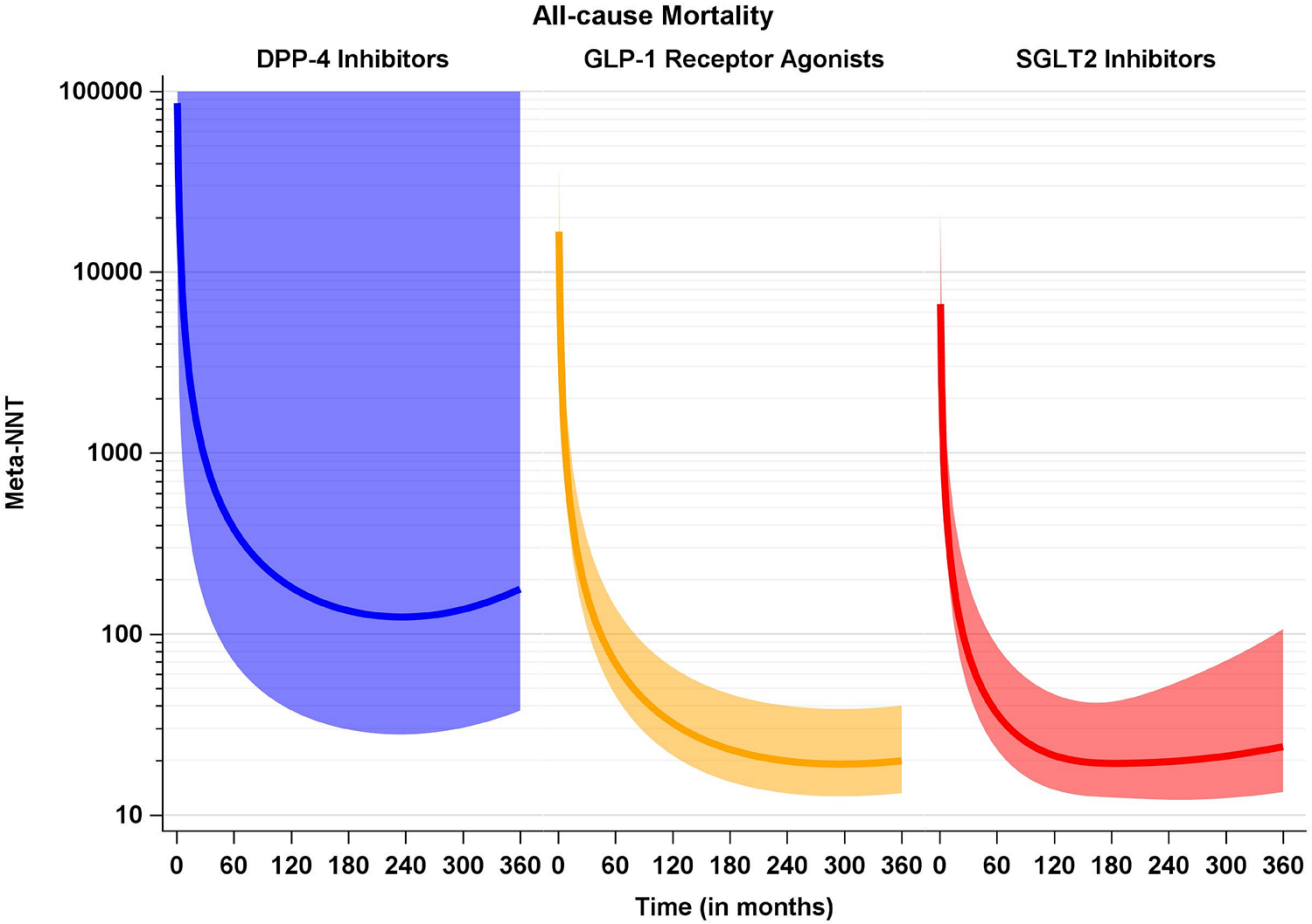
Projected overall Meta-NNTs for all-cause mortality, separated by drug classes (blue: DPP-4 inhibitors, yellow: GLP-1 receptor agonists, red: SGLT2 inhibitors) with their pointwise 95% confidence intervals. Estimates and confidence intervals are truncated from above at 100.000. Please note the logarithmic scale on the y-axis. With respect to the primary outcome, Meta-NNTs were computed from the single trials’ primary outcomes which slightly differ in their definition (see Table 1) (Color figure online)

Considering the lifetime perspective of treatment, Fig. 4 gives the projected Meta-NNTs in the three drug classes. Because the probability of death naturally increases in the time course and in both treatment groups, these Meta-NNTs achieve a minimum, that is, a maximum treatment effect, but increase after having reached this minimum. That is, even when assuming a constant treatment effect, Meta-NNTs do not have a monotonically decreasing time course.

In supplemental Fig. 2, we give scatterplots to compare the originally reported hazard ratios to those from the fitted Weibull models on the digitalized data. As can be seen, the correspondence is excellent. In terms of the intra-class correlation, this was 99.8% (95%-CI: 99.5%, 100%) for the primary outcome, and 99.5% (95%-CI: 98.9%, 100%) for all-cause mortality, where the upper limit of the confidence interval had been truncated at 100%.

In supplemental Fig. 3, we show the fit of the Weibull models to the extracted data by giving the Kaplan–Meier estimates in the two treatment groups for each outcome and each trial together with the 95% confidence intervals of the fitted Weibull survival functions. Again, there are no relevant differences that might compromise computation or interpretation of NNTs.

## Discussion

Treatment effects in the large CVOTs of new antidiabetic drugs look less impressive if they are reported as numbers needed to treat (NNTs) instead of hazard ratios. For example, the overall median hazard ratio across all trials for the two outcomes under study were found to be 0.87 here in favour of the trial drug. This corresponds to a hazard reduction of 13% for both outcomes and one might be tempted to interpret that one out of 13 or every 8^th^ (because 13% is roughly an eighth) benefits from treatment [9]. This is clearly an overestimation, instead and as shown in Fig. 2, 100 patients have to be treated for 29 months (the median follow-up time across all trials) to avoid one single event of the primary outcome, and 128 patients have to be treated for 39 months to avoid one single death.

Of course, the perceived overestimation of treatment effects by hazard ratios is especially large here due to the overall low number of events in the CVOTs, that is, the low baseline risk (displayed as the proportion of events in Table 2) for the outcomes in the placebo groups.

In view of these considerable differences between relative and absolute treatment effects, it is no surprise that trial authors and sponsors do not actively communicate NNTs, and of course, the FDA did not insist on that in their 2008 guideline. Exceptions are found in CREDENCE [27] and DAPA-CKD [28], where NNTs are reported in the main paper, LEADER [29] and EMPA-REG OUTCOME [30] report NNTs in follow-up papers.

Until the trials’ maximum observation times, we observe NNT courses to be largely decreasing, thus pointing to increasing treatment effects. It is tempting to speculate that this increase would last also with larger observation/treatment times. However, this is not the case, the NNT by definition (and at least for the outcome of mortality which in the long run would occur for every patient) will reach a minimum and increase again thereafter. This is true even under the two assumptions of a constant treatment effect and all patients staying on their initial treatment. We observe this behaviour of a first-increasing-then-decreasing treatment effect also in our NNT projection for the CVOT data which confirms again the validity of our approach. But of course, the results of these projections rely heavily on extrapolating beyond the trials’ observation times, and thus should be considered exploratory [10].

We restricted our study to the two outcomes reported here because one was the primary outcome as suggested by the FDA (see Table 1 for the slightly different definitions of the primary outcome in the respective trials) and the other (all-cause mortality) is the most unbiased clinical endpoint possible [31]. Moreover, all-cause mortality is the only outcome in the CVOTs that is not affected by competing risks.

There have been previous analyses of NNTs in the large CVOTs in type 2 diabetes [10, 18] where both research groups also relied on individual data extracted from the original publications. However, as compared to Davies et al. [18], we did not restrict to the class of GLP-1 receptor agonists, but report NNTs also for two other drug classes. Moreover, we also report on the additional outcome of all-cause mortality and offer an overall as well as a drug class-specific meta-analytic summary. Ludwig et al. [10] only report on a limited non-systematic sample of CVOTs in two drug classes and only give NNT estimates at the respective median follow-up time although they emphasize correctly that there is not a single global NNT for a specific trial. In addition, Ludwig et al. [10] do not use the original data (with the additional option to validate the digitalized data against those from the original publication), but rely on formulas for summary data. Finally, Davies et al. [18] and Ludwig et al. [10] use different software tools for data extraction and different statistical models to arrive at NNT estimates as compared to our approach. However, differences between reported NNTs (see supplemental Table 1) are marginal thus confirming in principle the consistency and validity of the approach of digital extraction of individual patient data from trial publications.

With respect to comparing originally reported hazard ratios and those computed from Weibull models within our own study, differences were also negligible. As such, it is also no disadvantage that the functional form of the NNT dynamics in time and all the derived NNT at fixed time points reported here were determined by the parametric form. It might rather be considered an advantage that a smooth and plausible function was generated. Moreover, we saw that the full Weibull survival functions (from which the NNTs are directly derived) give excellent fits to the digitalized survival data. There are also other possible parametric assumptions for the outcome data (e.g. Davies et al. [18] used the Royston–Parmar model which allows for even more flexible survival functions), but we chose the Weibull because it allows for computing hazard ratios and thus a comparison to the digitalized data. It should be also noted that the original hazard ratios from the CVOTs were all computed from Cox models that assume proportional hazards in treatment groups across the trial course, and of course, this proportional hazards assumption in the original trials might also be wrong. In effect, we feel the Weibull assumption is not that more restrictive than the proportional hazards assumption; however, a parametric model additionally allows for directly estimating survival probabilities and NNTs.

It is fair to point to some limitations of our study. We did not perform a lege artis systematic review with a systematic literature search and a published study protocol. Instead, we relied on a published scheme of the large CVOTs, which we yet consider complete. Indeed, we have waived a formal systematic review because there are a number of systematic reviews of the new antidiabetic drugs already available and the focus of our work was a methodical one, that is, the computation of absolute effects and their comparison with the reported relative effects.

Of course, meta-analyses of absolute effects face the same threats as those for all other outcome types, with the most important question always being whether the studies are sufficiently homogeneous to be pooled. Admittedly, in the case of absolute effects there is the additional point that effect estimates depend on the underlying baseline risk of the event. However, the CVOTs that we use here had been pooled in numerous meta-analyses up to now, thus confirming that researchers in general judge clinical heterogeneity to be small.

In terms of the NNT as an effect measure, Hansen et al. [32] noted the problem of the “lottery-like” appearance of the NNT. Communicating a fixed value for the NNT (for example, 100, as calculated here for the global NNT with respect to the primary outcome) seems to imply that only exactly one in 100 patients benefits from treatment. While this is formally true for the primary outcome, it is clinically far more plausible that most patients will benefit from treatment, at least to some extent, because anti-glycaemic treatment lowers glucose for most patients and glucose lowering is clearly correlated with the risk of a cardiovascular outcome. Finally, there is solid empirical evidence that patients have problems with interpreting and understanding NNTs. Indeed, even largely different NNT values presented to randomized groups do not result in different acceptance proportions to treatment [33–35]. We therefore agree with other researchers [36, 37] that the NNT should better not be used for communication with patients, but rather in research contexts and for communication with health professionals. However, it should be realized that relative effect measures like hazard ratios are also not well understood by patients [38].

All included trials address new antidiabetic drugs, however, no other factors that also determine total cardiovascular risk (e.g. blood pressure, cholesterol, environmental background, genetic background, etc.). In addition, reporting of cardiovascular risk of trial populations was limited. Study designs with multifactorial therapeutic approaches and careful characterization of the underlying population risk (e.g. the NID-2 study [39]) are probably more appropriate to evaluate absolute treatment effects. However, our approach yields results at an average risk of all included patients and is thus a valid way to describe absolute treatment effects.

In summary, this study provides a comprehensive analysis of the absolute treatment effects of the newer antidiabetic drug classes (DPP-4 inhibitors, GLP-1 receptor agonists, and SGLT2 inhibitors) from the large CVOTs of new antidiabetic drugs. We found that the respective treatment effects look much less impressive when communicated on an absolute scale, as numbers needed to treat. For a valid overall picture of the benefit of these new antidiabetic drugs, trial authors should thus also report treatment effects on an absolute scale. Authorities responsible for approval should continue to ask for absolute effects estimates to enable health professionals and policy makers to make better informed decisions.

## Supplementary Information

Below is the link to the electronic supplementary material.

Supplementary file1 (DOCX 911 kb)
