# Supplementary material for: Absolute treatment effects for the primary outcome and all-cause mortality in the cardiovascular outcome trials of new antidiabetic drugs: a meta-analysis of digitalized individual patient data
Source: Acta Diabetol. 2022 Jul 25;59(10):1349–59. doi: 10.1007/s00592-022-01917-9 (PMC9402762; doi:10.1007/s00592-022-01917-9)
Supplement: Supplementary file 1 — Supplementary file1 (DOCX 911 kb) [file 592_2022_1917_MOESM1_ESM.docx]

Supplemental Figure 1: Kaplan-Meier estimates and estimated Weibull survival functions for the outcome all-cause mortality from the EMPA-REG trial


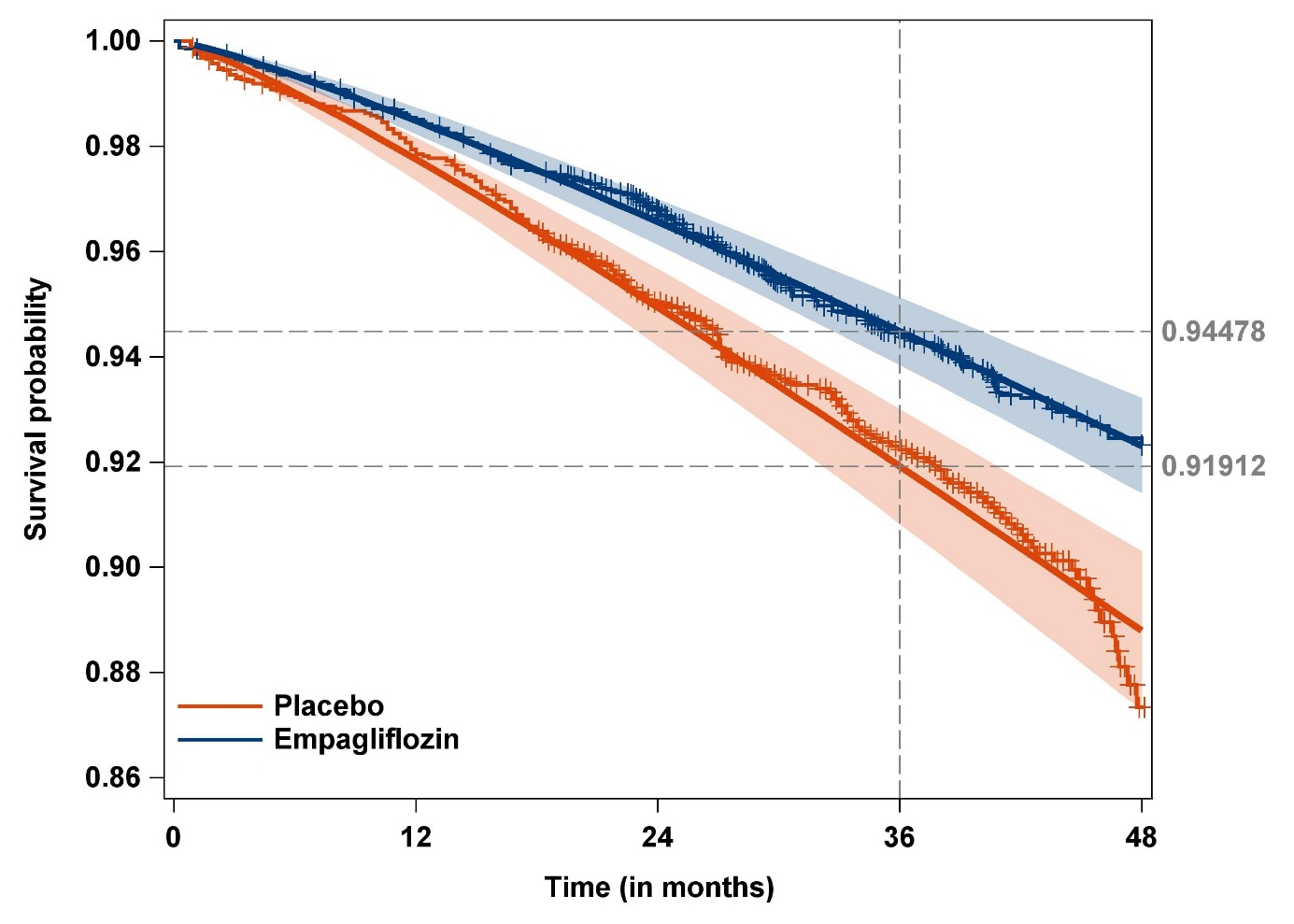


As an example for the computation of a single NNT from the available digitalized data, we refer to the figure above. This displays the two Kaplan-Meier estimates for the outcome all-cause mortality from the EMPA-REG trial with the respective estimated Weibull survival functions and their 95% confidence intervals. To arrive at the reported NNT of 39 (see table 3 in the main paper) we first subtract the two estimated event probabilities after 36 months (p=0.94478 in the empagliflozin group and p=0.91912 in the placebo group) and find a risk difference of 0.94478 - 0.91912 = 0.02566 which is the length of the black arrow in the figure. Inverting this risk difference (NNT = 1 / RD = 1 / 0.02566 = 38.97116) yields the reported NNT which is then further processed in the meta-analyses.

Supplemental Figure 2: Scatterplots to compare the hazard ratios from the original publications (x-axis) with the hazard ratios from the fitted Weibull model of the extracted data (y-axis). Left hand side: Hazard ratios for the primary outcome, Right hand side: Hazard ratios for all-cause mortality


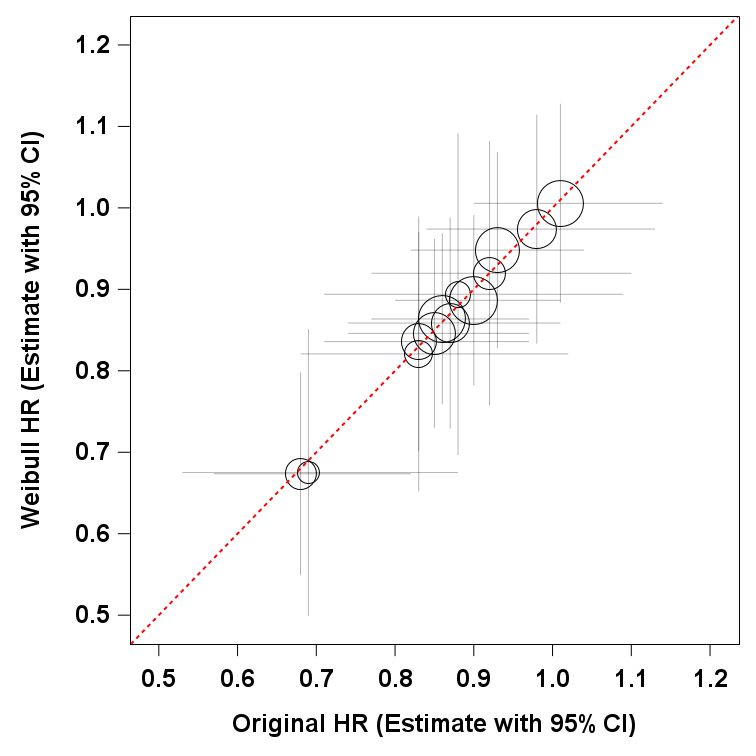

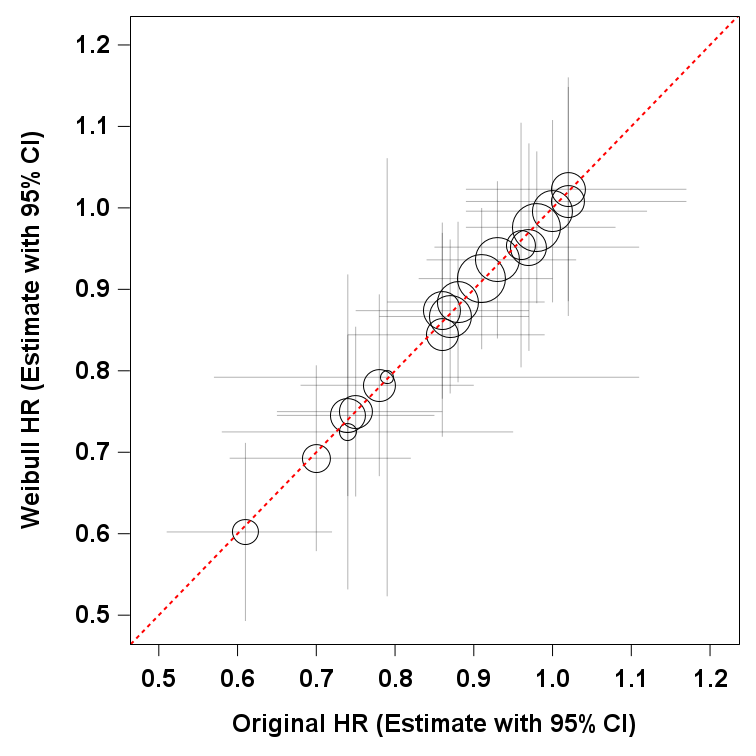


Supplemental Figure 3: Kaplan-Meier estimates of the extracted data with 95% confidence intervals of the Weibull fit for the respective maximum observation time of the trial


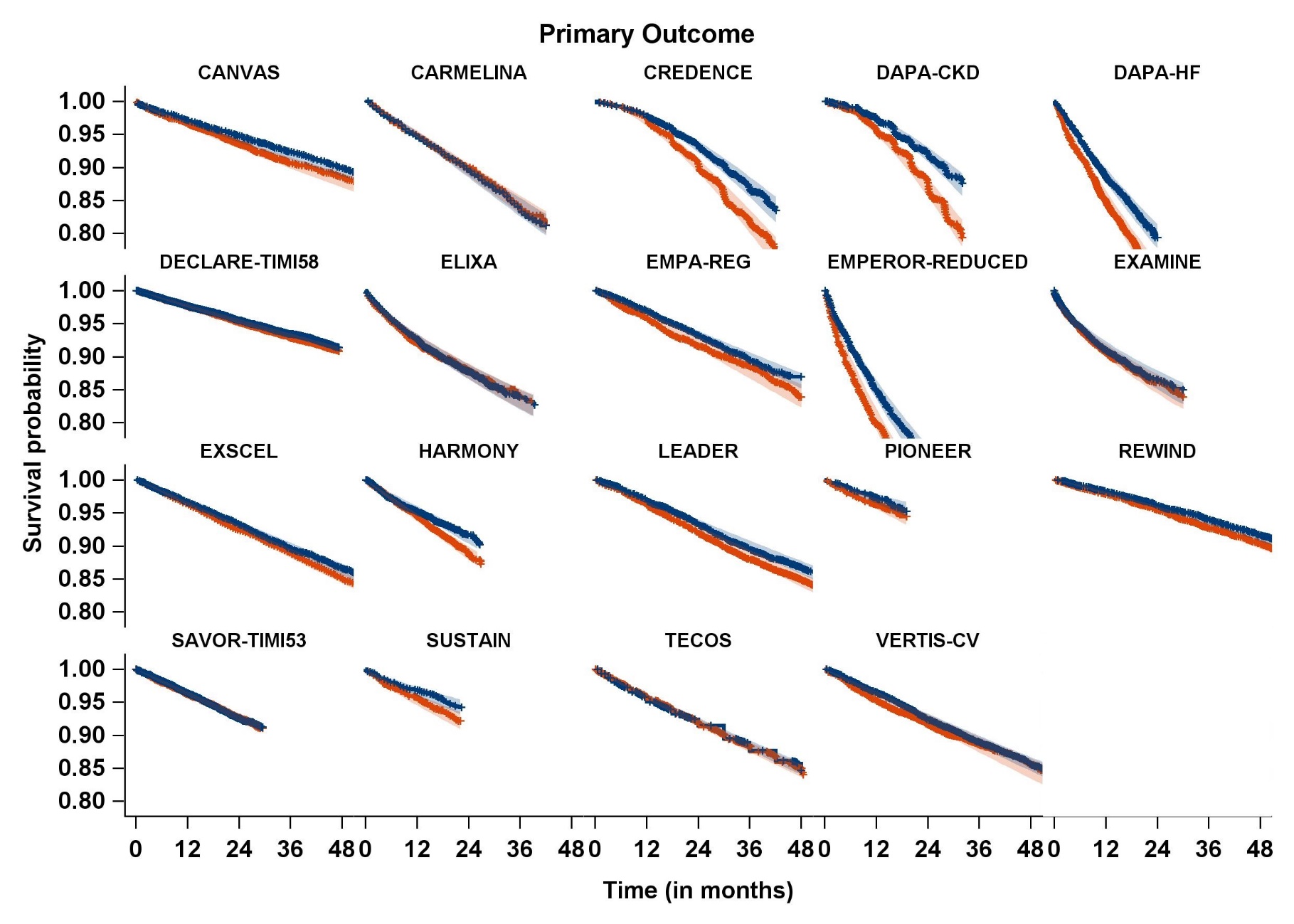


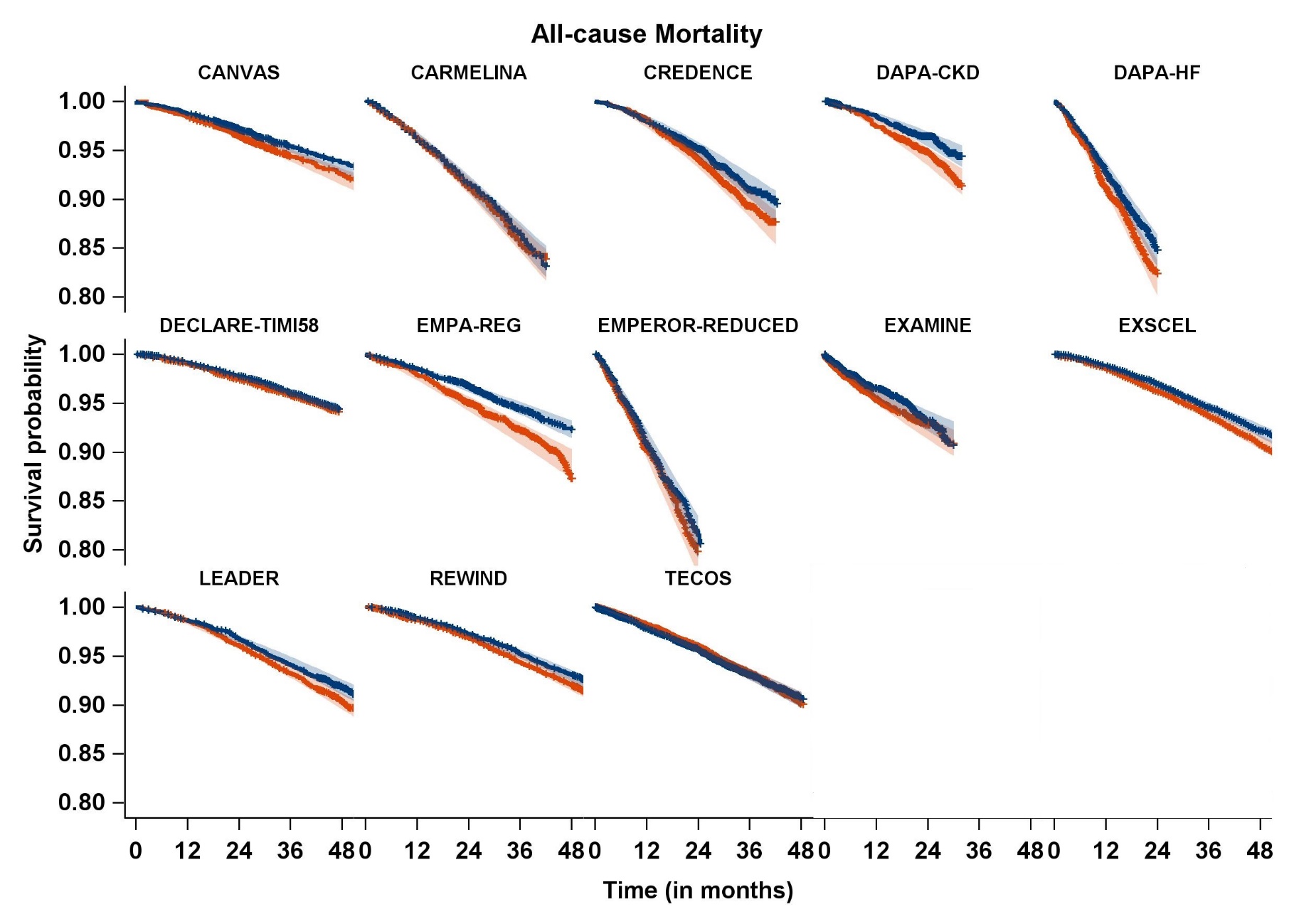


Supplemental Table 1: NNTs for the primary outcome from Davies et al. [18] and Ludwig et al. [10] in comparison to the NNTs computed by us.

| **Trial** | **Time point (years)** | **Reported NNT (95% CI)** | **Our NNT (95% CI)** |
| --- | --- | --- | --- |
|  | **Davies et al.** | |  |
|  | | | |
| EXSCEL | 1 | 365 [−71, 801] | 326 [159, -7005] |
| HARMONY | 1 | 83 [35, 131] | 83 [52, 197] |
| LEADER | 1 | 209 [53, 366] | 207 [117, 881] |
| PIONEER | 1 | 135 [−61, 331] | 136 [55, -299] |
| REWIND | 1 | 429 [41, 816] | 434 [227, 4938] |
| SUSTAIN | 1 | 88 [15, 161] | 80 [44, 464] |
|  | | | |
| REWIND | 3 | 129 [13, 246] | 127 [67, 1366] |
| EXSCEL | 3 | 126 [−24, 275] | 110 [54, -2433] |
| HARMONY | 3 | 30 [13, 48] | 30 [19, 72] |
| LEADER | 3 | 65 [17, 113] | 69 [39, 291] |
| PIONEER | 3 | 50 [−23, 124] | 50 [20, -108] |
| REWIND | 3 | 129 [13, 246] | 127 [67, 1366] |
| SUSTAIN | 3 | 31 [6, 57] | 33 [18, 188] |
|  | | | |
|  | **Ludwig et al.** | |  |
|  | | | |
| HARMONY | 1.6 | 53 [36, 116] | 53 [34, 126] |
| LEADER | 3.8 | 56 [33, 243] | 56 [32, 234] |
| REWIND | 5.1 | 67 [38, 803] | 73 [38, 775] |
| SUSTAIN-6 | 2.1 | 45 [28, 235] | 43 [24, 248] |
| DECLARE-TIMI58 | 4.2 | 104 [66, 355] | 169 [66, -300] |
| EMPA-REG | 3.1 | 63 [34, 882] | 55 [29, 528] |
